# Observation of ionic conductivity on PUA-TBAI-I_2_ gel polymer electrolyte

**DOI:** 10.1038/s41598-021-03965-7

**Published:** 2022-01-07

**Authors:** K. L. Chai, Min Min Aung, I. M. Noor, H N Lim, L C Abdullah

**Affiliations:** 1 Higher Education Centre of Excellence (HiCoE), Institute of Tropical Forestry and Forest Products, Universiti Putra Malaysia (UPM), 43400 Serdang, Selangor Malaysia; 2 Department of Chemistry, Faculty of Science, Universiti Putra Malaysia (UPM), 43400 Serdang, Malaysia; 3 Unit Chemistry, Centre of Foundation Studies for Agricultural Science, Universiti Putra Malaysia (UPM), 43400 Serdang, Selangor Malaysia; 4 Physics Division, Centre of Foundation Studies for Agricultural Science, Universiti Putra Malaysia (UPM), 43400 Serdang, Selangor Malaysia; 5 Material Synthesis and Characterization Laboratory, Institute of Advanced Technology, Universiti Putra Malaysia (UPM), 43400 Serdang, Selangor Malaysia; 6 Department of Chemical and Environmental Engineering, Faculty of Engineering, Universiti Putra Malaysia (UPM), 43400 Serdang, Malaysia

**Keywords:** Renewable energy, Materials chemistry, Polymer chemistry

## Abstract

Jatropha oil-based polyurethane acylate gel polymer electrolyte was mixed with different concentrations of tetrabutylammonium iodide salt (TBAI). The temperature dependences of ionic conductivity, dielectric modulus and relaxation time were studied in the range of 298 to 393 K. The highest ionic conductivity of (1.88 ± 0.020) × 10^–4^ Scm^−1^ at 298 K was achieved when the gel contained 30 wt% of TBAI and 2.06 wt% of I_2_. Furthermore, the study found that conductivity-temperature dependence followed the Vogel-Tammann Fulcher equation. From that, it could be clearly observed that 30 wt% TBAI indicated the lowest activation energy of 6.947 kJ mol^−1^. By using the fitting method on the Nyquist plot, the number density, mobility and diffusion coefficient of the charge carrier were determined. The charge properties were analysed using the dielectric permittivity, modulus and dissipation factor. Apart from this, the stoke drag and capacitance were determined.

## Introduction

Concern for the environment and the dependency on fossil fuel for producing chemical feedstock has researchers focusing their effort into developing materials which use green and sustainable materials. Sustainably grown crops such as *Jatropha curcas* plant that are easily cultivated^1^, as well as being hardy, and non-edible^2,3^ in nature with a high yield of oil per unit hectare^4–6^ help spearhead the research into discovering more applications in the field of electrochemistry^7,8^.

Deriving a polyurethane acrylate (PUA) polymer electrolyte from jatropha oil produces a gel like material which is soft and sticky. A gel based bio polymer electrolyte provides a fair amount of advantage compared to their liquid and solid electrolyte counterparts^9,10^. Being in a gel like state, it combines the advantage of a solid electrolyte such as high safety as there is no leakage, with low flammability, flexibility, processability and thermal stability^11,12^ at the same time offering some of the benefits of a liquid electrolyte without the drawbacks. As the electrolyte is sticky in nature, it does not spill easily a like liquid electrolyte. It also can operate at higher temperatures^13,14^ as it does not face an issue with solvent evaporation such as occurs with a liquid electrolyte^15–17^. It is also flexible in nature which offers great mechanical properties over a solid electrolyte^13,14^. It also generally has a higher ionic conductivity compared to a solid electrolyte^10,18,19^.

Due to the advantages mentioned above, jatropha oil-based PUA is used as the host polymer for preparing the gel polymer electrolyte. This is because PUA has high cross-linking density, solvent resistance, good mechanical properties and excellent adhesion^20^. Other than that, PUA has a unique structure in which it has hard and soft segments that are arranged in an alternative pattern on the same chain^21–23^. With this unique property, PUA acts as a suitable candidate for electrochemical devices^23–27^.

However, it is known that the ionic conductivity of gel polymer electrolytes is still lower than liquid electrolytes. Therefore, further in-depth study for the feasibility of PUA-TBAI-I_2_ gel polymer electrolyte must be conducted. The PUA-TBAI-I_2_ gel polymer electrolyte performance can be gauged by extensive study of its electrochemical properties such as the behaviour of the charge carriers, dielectric permittivity, ionic conductivity, electrical modulus, relaxation time, number density, including the mobility and diffusion coefficient of the charged carriers. To gauge the feasibility of the Gel Polymer Electrolyte (GPE) for industrial usage, the above electrochemical properties can also be measured at various temperatures.

In this work, a PUA host polymer was added with various concentrations of tetrabutylammonium iodide (TBAI) salt in order to enhance the ionic conductivity. TBAI salt was used together with iodine to form the redox I^−^/I_3_^−^ redox shuttle. TBAI salt was chosen as the primary salt in this gel polymer electrolyte system. This was due to its low lattice energy of 320.8 kJ mol^−1^^28^. The low lattice energy makes it easier to dissociate into ions compared to other iodide salts. The higher degree of dissociation of iodide ions increases the number density of the charge carriers which also directly increase the mobility of the charge carriers. This results in the increase of ionic conductivity of the GPE. This phenomena has been reported by Bandara et al*.*^29^. Their sample containing 0.1 g polyacrylonitrile (PAN): 0.4149 g ethylene carbonate (EC): 0.3848 g propylene carbonate (PC): 0.0696 g TBAI: 0.0048 g iodine (I_2_) recorded an ionic conductivity of 2.14 × 10^–3^ S cm^−1^^29^. Chowdhury et al.^30^ reported the highest room temperature ionic conductivity at 3.46 × 10^–3^ S cm^−1^ for their 0.27 g PAN:1.02 g EC: 1.02 g PC: 1 g TBAI: 0.069 g I_2_ gel polymer electrolyte. The values of ionic conductivity in^29,30^ increased about eleven orders of magnitude compared to pure PAN which was ≤ 10^–14^ S cm^−1^^31^. Another example was reported by Theerthagiri et al*.*^32^. Their sample of polyvinylidene fluoride (PVDF): poly(methyl methacrylate) PMMA: EC: dimethylformamide (DMF): 60% TBAI: I_2_ polymer electrolyte achieved an ionic conductivity value of 5.11 × 10^–3^ S cm^−1^ at 303 K. Tiong et al*.* reported the same findings^33^. Their gel polymer electrolyte with a composition of 7.02 wt% PVA: 7.02 wt% polyethylene glycol (PEO): 30.11 wt% EC: 30.00 wt% dimethyl sulfoxide (DMSO): 24.08 wt% TBAI: 1.66 wt% I_2_ recorded the highest ionic conductivity of 5.50 × 10^–3^ S cm^−1^ at room temperature. From the literature above, it is known that TBAI salt is suitable to be used as a primary salt in a PUA gel polymer electrolyte system.

## Experimental

### Materials

**Table:** 

Name	Chemical structure	Company
Jatropha oil (JO)		Bionas Biofuel Sdn Bhd., Kuala Lumpur, Malaysia
Formic acid (99% purity)		R&M Chemical, Malaysia
Sulphuric acid		R&M Chemical, Malaysia
Elemental iodine		R&M Chemical, Malaysia
Dimethylformamide (DMF)		R&M Chemical, Malaysia
Methanol		HmbG^®^ Chemical, Malaysia
Aqueous hydrogen peroxide (30% purity)		Merck, Germany
2-Hydroxyethyl 2-methylpop-2-enoate (HEMA) with 80% technical grade		Sigma Aldrich, Germany
Tetrabutylammonium iodide (TBAI)		Sigma Aldrich, Germany
2,4-Diisocyanato-1-methylbenzene (TDI) at 80% technical grade		Acros Organics, New Jersey

### Method

#### Preparation of polyurethane acrylate (PUA)

Jatropha oil-based PUA was synthesised by a three-step process: epoxidation, hydroxylation and the introduction of the acrylate group in the urethanation process^27,34^. During the epoxidation process, the jatropha oil was pre-mixed with formic acid under mechanical stirring using an overhead stirrer at 313 K. Hydrogen peroxide was added slowly into the mixture using a dropping funnel to prevent over heating of the mixture solution. The mixture was then heated to 333 K while being stirred continuously for 6 h. After 6 h, the mixture was left to cool down to room temperature before transferring it to a separating funnel. The mixture was allowed to settle in the separating funnel and the aqueous layer was removed. The remaining organic layer was rinsed with warm distilled water several times to remove any excess acid. Thus the epoxidised jatropha oil (EJO) was produced.

After that, the hydroxylation process was carried out. Some 133 g of methanol, 0.93 g of sulphuric acid and 15 g of distilled water was pre-mixed and stirred for about 15 min at 313 K. Then, 150 g of EJO was added to the mixture and was heated up to 338 K for another 30 min. The polyol was allowed to cool down to room temperature before it was poured into a separating funnel. After that, the excess methanol and distilled water in the polyol was removed by using a vacuum rotary evaporator until a clear yellow polyol was obtained.

Polyol, TDI, HEMA and DMF as solvent were used in order to prepare polyurethane acrylate. An amount of 20 g of Polyol and 10 ml of DMF were mixed at 323 K, then 8.57 g of TDI was added drop wise into the mixture using a dropping funnel. The mixture was then heated up to 333 K for about 2 h. During this process, 10 ml of DMF was added into the mixture. After that, the mixture was allowed to cool to 313 K and 6.38 g of HEMA was added drop wise. The mixture was heated up again to 323 K for another 1 h. At the same time, another 5 ml of DMF was added. This was done to reduce the viscosity of the mixture and the PUA gel polymer electrolyte was successfully produced.

#### PUA-TBAI-I_2_ gel polymer electrolyte preparation

PUA-TBAI-I_2_ gel polymer electrolytes were prepared with the selected ratio as listed in Table 1. The TBAI salt was dissolved in PUA in a closed glass container and was stirred at room temperature. I_2_ chips (10 mol% of iodide salt) were then added into the mixture and stirred overnight. The electrolytes were placed in a desiccator for 24 h before being used for characterisation.

**Table 1:** Composition of PUA-TBAI-I_2_ gel polymer electrolyte.

PUA:TBAI (wt%:wt%)	PUA (g)	TBAI (g)	I_2_ (g)
90:10	1.500	0.1679	0.0115
80:20	1.500	0.3816	0.0262
70:30	1.500	0.6624	0.0455
60:40	1.500	1.0479	0.0720
50:50	1.500	1.6104	0.1107

### Characterisation

#### Electrochemical impedance spectroscopy (EIS)

EIS measurements were carried out to study the resistance and capacitance properties of the electrolytes. A HIOKI IM3570 Impedance Analyser was used to perform the measurements at various temperatures ranging from 298 to 393 K. The PUA-TBAI-I_2_ GPE was sandwiched in air between two stainless steel electrodes with a 2.011 cm^2^ working area equipped with a 0.26 cm thickness separator and then the assembly was placed into the coin cell and clamped. The coin cell was placed in the oven using controlled heating for the conductivity-temperature studies. A small sine wave with voltage of 10 mV with a frequency range of 50 Hz to 1 MHz was applied across the sample. A current flow in the sample was recorded and the impedance data was calculated from it. Nyquist plots were plotted based on the real (*Z*_*r*_) and negative imaginary (− *Z*_*i*_) impedance data. The intercept of the plot on the real axis corresponded to the bulk resistance (*R*). At lower temperatures, *R* was obtained from the intersection of semicircle and the spike. At higher temperatures, the semicircle disappeared as the resistance of the sample became smaller and smaller until only the spike was observed. Then, the *R* was determined from the intercept on the *Z*_*r*_ axis. The ionic conductivity (*σ*) of the electrolyte was then calculated by using the equation below^35,36^:

1 $$\sigma = \frac{t}{R \times A}$$

In the above equation, *t* is sample thickness and *A* is the electrolyte/electrode contact surface area.

For various temperatures, the equations for *Z*_*r*_ and − *Z*_*i*_ impedances are as follows:

2 $$Z_{r} = R + \frac{{\cos (\frac{{\pi p_{2} }}{2})}}{{k_{2}^{ - 1} \omega^{p2} }}$$

3 $$Z_{i} = \frac{{\sin (\frac{{\pi p_{2} }}{2})}}{{k_{2}^{ - 1} \omega^{{p_{2} }} }}$$

Here, *k*_2_^–1^ is the electrical double layer capacitance at the electrode/electrolyte interface. *P*_*2*_ is the angle between the Z′ axis and the spike line. *ɷ* is the angular frequency corresponding to the minimum imaginary impedance. From the fitting parameters, n, µ, and D could be determined using Eqs. (4) to (7).

4 $$D = D_{0} \exp \{ - 0.0297[Ln(D_{0} )]^{2} - 1.4348[Ln(D_{0} )] - 14.504\}$$

5 $$\mu = \frac{{eD_{0} \exp \{ - 0.0297[Ln(D_{0} )]^{2} - 1.4348[Ln(D_{0} )] - 14.504\} }}{{k_{b} T}}$$

6 $$n = \frac{{\sigma k_{b} T}}{{e^{2} D_{0} \exp \{ - 0.0297[Ln(D_{0} )]^{2} - 1.4348[Ln(D_{0} )] - 14.504\} }}$$

7 $$D_{0} = \frac{{4k^{4} d^{2} }}{{R^{4} \omega^{3} }}$$

The value of the diffusion coefficient could then be calculated by using the Stokes–Einstein equation:

8 $$D = \frac{{\mathop K\nolimits_{b} T}}{6\pi \eta r}$$

The formula for Stoke drag is shown as below:

9 $$\mathop F\nolimits_{d} = 6\pi r\eta v$$

By substituting Eq. (14) into Eq. (13), the Stoke drag equation can be obtained:

10 $$\mathop F\nolimits_{d} = \frac{{\mathop K\nolimits_{b} T}}{D}$$

where *K*_*b*_ is the Boltzmann constant, *T* is the absolute temperature, η is viscosity, r is radius and v is velocity. In this work, v can be ignored as v is kept constant.

To further understand the ionic conductivity behaviour in PUA-TBAI-I_2_ GPE, the complex permittivity was studied. The equations below were used to calculate the complex permittivity of the electrolyte^37,38^:

11 $$\varepsilon_{r} = \frac{{Z_{i} }}{{\omega C_{o} \left( {Z_{r}^{2} + Z_{i}^{2} } \right)}}$$

12 $$\varepsilon_{i} = \frac{{Z_{r} }}{{\omega C_{o} \left( {Z_{r}^{2} + Z_{i}^{2} } \right)}}$$

Here $C_{o} = \frac{{\varepsilon_{o} A}}{t}$ where $\varepsilon_{o}$ is the permittivity of free space. *ω* = 2π*f* where *f* is the frequency in Hz. For complex electrical modulus studies, the real electrical modulus, *M*_*r*_ and imaginary electrical modulus, *M*_*i*_ can be achieved by using the following equation:

13 $$M_{r} = \frac{{\varepsilon_{r} }}{{\left( {\varepsilon_{r}^{2} + \varepsilon_{i}^{2} } \right)}}$$

14 $$M_{i} = \frac{{\varepsilon_{i} }}{{\left( {\varepsilon_{r}^{2} + \varepsilon_{i}^{2} } \right)}}$$

15 $$\tan \delta = \frac{{M_{i} }}{{M_{r} }}$$

## Results and discussion

### Room temperature conductivity

Table 2 lists the parameters of the ionic conductivity with various concentration of TBAI salt that were added into the PUA gel polymer electrolyte at room temperature. From Table 2, the ionic conductivity of 10 wt% TBAI salt was calculated to be (1.40 ± 0.003) × 10^–4^ S cm^−1^ using an *R* value of (922 ± 2) Ω. On the other hand, 30 wt% TBAI salt showed the lowest R value of (686 ± 7) Ω and a calculated ionic conductivity value of (1.88 ± 0.020) × 10^–4^ S cm^−1^. This was due to R which was inversely proportional to the ionic conductivity as in Eq. (1).

**Table 2:** Parameters of ionic conductivity with various weight percentages of TBAI salt added at room temperature.

TBAI salt (wt%)	Resistance, R (Ω)	Number density, n (× 10^21^ cm^−3^)	Mobility, µ (× 10^–7^ cm^2^ V^−1^ s^−1^)	Diffusion coefficient, D (× 10^–8^ cm^2^s^−1^)	Ionic conductivity, σ (× 10^–4^ S cm^−1^)
10	922 ± 2	1.68 ± 0.071	5.21 ± 0.223	1.34 ± 0.057	1.40 ± 0.003
20	749 ± 6	1.84 ± 0.025	5.94 ± 0.144	1.53 ± 0.037	1.76 ± 0.033
30	686 ± 7	1.93 ± 0.042	6.24 ± 0.117	1.60 ± 0.030	1.88 ± 0.020
40	838 ± 4	2.73 ± 0.045	3.53 ± 0.052	0.91 ± 0.013	1.54 ± 0.007
50	885 ± 7	3.46 ± 0.075	2.66 ± 0.075	0.68 ± 0.019	1.47 ± 0.019

Ionic conductivity is the measurement of free ions transported from one place to another inside the polymer matrix. The equation for Ionic conductivity is given as:

16 $$\sigma = n\mu e$$

**Figure 1:**
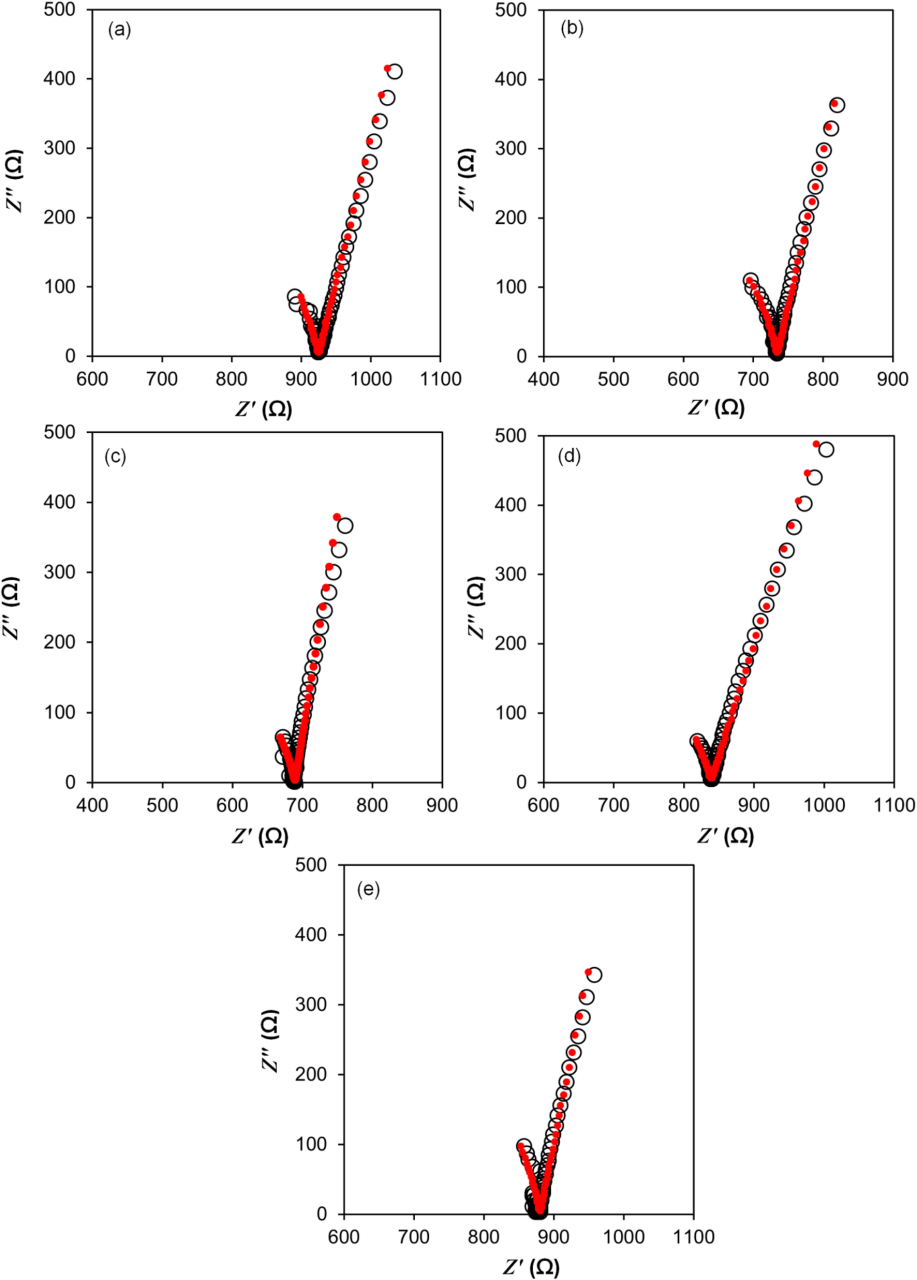
Nyquist plots of (**a**) 10 wt%, (**b**) 20 wt%, (**c**) 30 wt%, (**d**) 40 wt% and (**e**) 50 wt% TBAI salt based polyurethane acrylate gel polymer electrolyte at room temperature (open circle refers to experimental points and filled circle refers to fitted points).

Here *n* is the number density, *µ* is the mobility of charge carriers and *e* is the elementary charge. From Eq. (16), the values of *n* and *µ* are the two main parameters to determine the ionic conductivity. They can be estimated by fitting a Nyquist plot (Fig. 1) based on an equivalent circuit model^39^. The values of *n*, *µ* and *D* are tabulated in Table 2. Based on the *n*, *µ* and *D* values obtained from the fitting of the Nyquist plot, it was observed that the ionic conductivity increases from (1.40 ± 0.003) × 10^–4^ S cm^−1^ for 10 wt% TBAI salt to (1.88 ± 0.020) × 10^–4^ S cm^−1^ for 30 wt% TBAI salt. This suggests that the *n*, *µ* and *D* values increased as the amount of TBAI salt added into the PUA gel polymer electrolyte increased from 10 to 30 wt%.

In comparison with the 10 wt% TBAI salt sample and the 30 wt% TBAI salt sample, the value of *n*; (1.68 ± 0.071) × 10^21^ cm^−3^ increased to (1.93 ± 0.042) × 10^21^ cm^−3^. The value of *µ*; (5.21 ± 0.223) × 10^–7^ cm^2^ V^−1^ s^−1^ increased to (6.24 ± 0.117) × 10^–7^ cm^2^ V^−1^ s^−1^_._, while the value of *D* increased from (1.34 ± 0.057) × 10^8^ cm^2^ s^−1^ to (1.60 ± 0.030) × 10^8^ cm^2^ s^−1^. However, a TBAI salt loading of 40 wt% and above showed a lower ionic conductivity compared to the 30 wt% TBAI salt. For instance, the 50 wt% TBAI salt sample showed an ionic conductivity of (1.47 ± 0.019) × 10^–4^ S cm^−1^. This was mainly due to the decrease of the *µ* value of (2.66 ± 0.075) × 10^–7^ cm^2^ V^−1^ s^−1^ and the *D* value of (0.68 ± 0.019) × 10^8^ cm^2^ s^−1^ as compared to the 30 wt% TBAI salt. As the number density of the charge carriers increased, more salt dissociation occurred. The increase of salt dissociation tended to cause the *µ* and *D* values to decrease as a blocking effect occurs due to the presence of too many charged carriers in the electrolyte system. Ion-ion interaction occurred and this favoured the formation of ion clusters^15,40,41^. This has been shown in a study conducted by Kadir et al.^42^ where it was mentioned that the rate of ion dissociation was favourable at a concentration range of around 10 wt% to 30 wt%. At higher concentrations of salt, ion association took place and neutral charges were formed which in return reduced the mobility of free ions and resulted in a decrease in ionic conductivity. Another example was spotted in the report of Addullaziz Abdukarimov et al.^43^ who reported that as the amount of TPAI salts in PEO: PC: EC: DMF: I_2_ liquid electrolyte increased, the ionic conductivity increased up to 8.98 ± 0.23 mS cm^−1^ for a sample containing 19.61 wt% TPAI as it had the lowest R value (14.2 Ω). Addullaziz Abdukarimov et al. also stated that the value of the ionic conductivity could be enhanced by increasing the number density of the free ions in the liquid electrolytes.

Thus in this work, the R value is expected to decrease with the increase of TBAI salt. The increase of the number density and the mobility of free ions also contributed to the increase of ionic conductivity. As more TBAI salt was added to the GPE, ion dissociation increased accordingly which caused an increase in free ions present in the PUA polymer matrix. Due to the increase of free ions in the polymer matrix, this could lead to an increase in ion mobility which in return increased the ionic conductivity of the gel polymer electrolyte.

### Conductivity-temperature studies

The ionic conductivity of the electrolyte with respect to different temperatures ranging from 298 to 393 K was conducted in this study. The Nyquist plot acquired from the study was fitted using the best equivalent circuit model. The Nyquist plot obtained could appear in three forms: (i) a semicircle predominantly at low frequency, (ii) a spike at high frequency or (iii) a combination of both semicircle and a spike^44^. At low temperature, the Nyquist plot showed a combination of a semicircle and spike. As the temperature increased, the semicircle which appeared at lower frequencies disappeared and left only a spike at higher frequencies.

**Figure 2:**
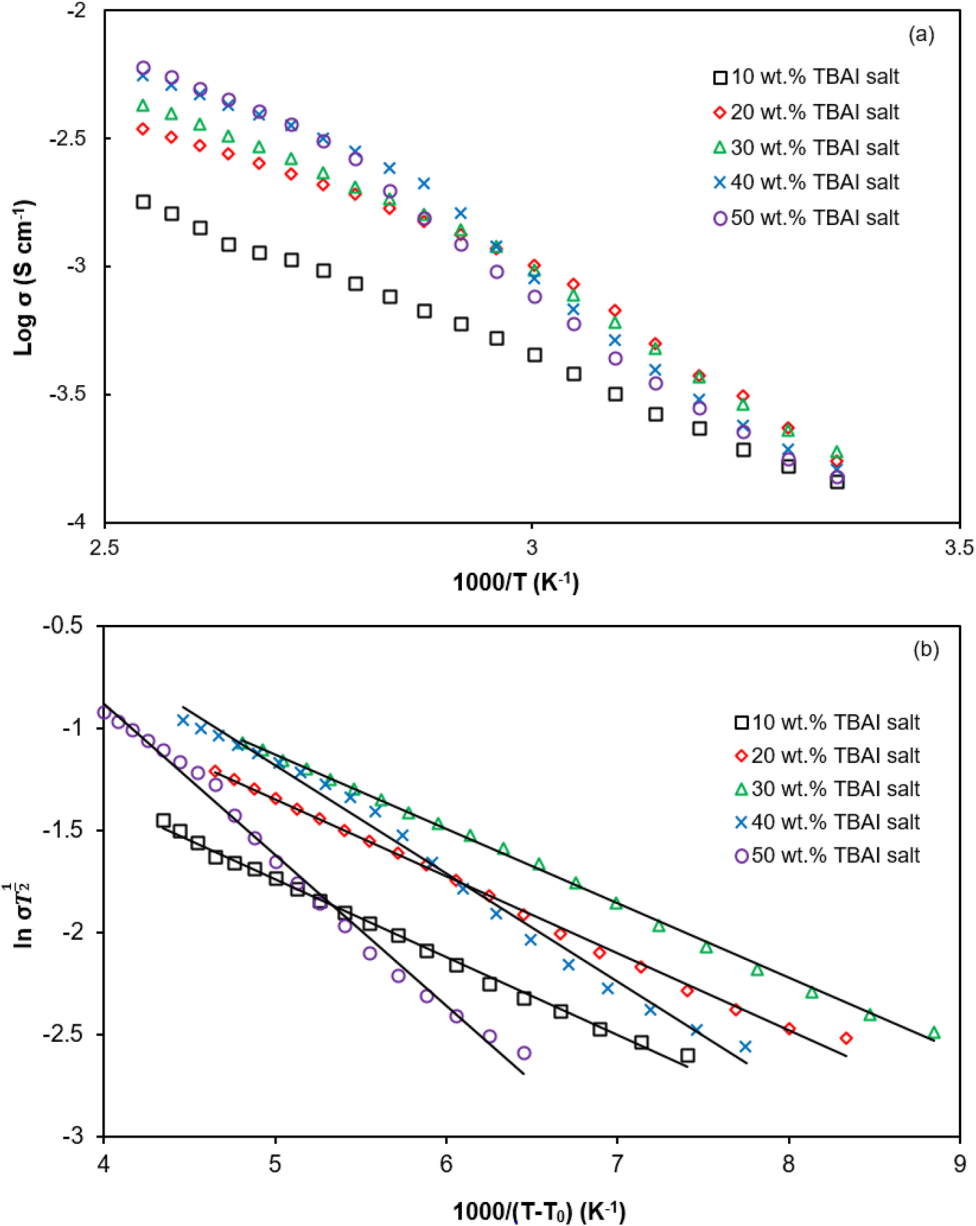
Temperature dependence of ionic conductivity followed (**a**) Arrhenius relationship and (**b**) VTF relationship for PUA-TBAI-I_2_ gel electrolyte systems.

The ionic conductivity of the PUA-TBAI-I_2_ gel electrolyte system at various temperatures is illustrated in Fig. 2. From Fig. 2a, it can be seen that the plot is not linear, but a curved plot is observed. This suggested that the temperature dependence of the ionic conductivity obeys the Vogel-Tammann Fulcher (VTF) relationship to interpret this behaviour. The VTF equation is as below:

17 $$\sigma T^{\frac{1}{2}} = A\exp \left[ {\frac{{ - E_{a} }}{{k_{b} \left( {T - T_{0} } \right)}}} \right]$$

Here *A* is the pre-exponential factor, *E*_a_ is pseudo-activation energy, *k*_*b*_ is the Boltzmann constant, *T* is absolute temperature and *T*_*o*_ is the ideal glass transition temperature (temperature at which the configurational entropy varnishes). Coupled with Fig. 2b, the value of *E*_*a*_ can be estimated from the gradient of Eq. (17). The values of *E*_*a*_ and *T*_*o*_ obtained from Fig. 2b are listed in Table 2.

**Figure 3:**
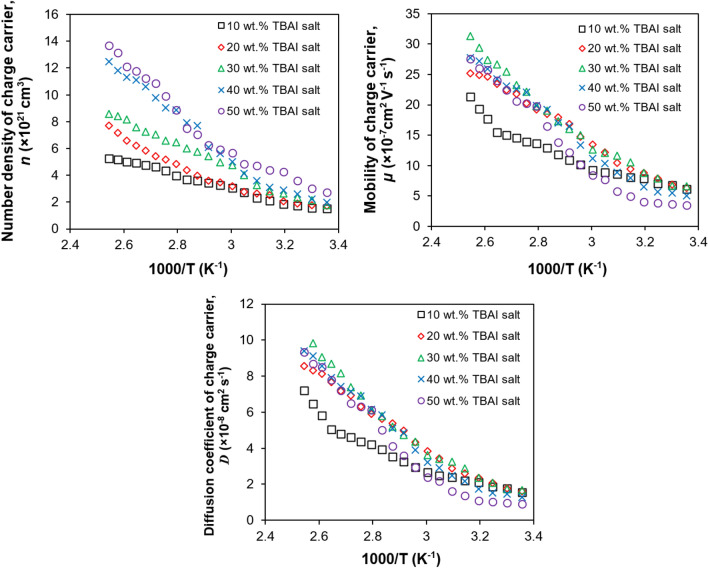
Number density (*n*), mobility (*µ*) and diffusion coefficient (*D*) of charge carriers at various temperatures.

Figure 2 shows that the ionic conductivity increases as the temperature increases. The increase of ionic conductivity is due to the increase of number density^41,45,46^ and the mobility of the charge carriers (proved in Fig. 3). When the temperature increases, the ions in the PUA-TBAI-I_2_ electrolyte will dissociate into cations (TBA^+^) and anions (I^−^) once they receive enough energy to break the bonds. This will produce more free ions available in the PUA-TBAI-I_2_ gel polymer electrolyte. This reason explains the increase of the number density of charge carriers with temperature as showed in Fig. 3.

In Table 3, it can be seen that the negative gradient for 30 wt% TBAI salt is the least which is − 0.364. This can be attributed to the low activation energy and segmental motion of the PUA polymer chain. The *T*_*o*_ and *E*_*a*_ values drop gradually from 10 wt% TBAI salt to 30 wt% TBAI salt. This was due to the movement of the free ions. The free ions move with acceleration from one site to another within the polymer matrix. Due to this, the 30 wt% TBAI salt recorded the lowest activation energy and glass transition temperature. Beyond 30 wt% TBAI salt, the E_a_ value increased from 6.947 to 14.183 kJ/mol for the sample containing 50 wt% TBAI salt.

**Table 3:** *T*_*o*_, *R*^2^, gradient and *E*_*a*_ for PUA-TBAI-I_2_ gel electrolyte systems.

TBAI (wt%)	*T*_*o*_ (K)	*R*^2^	Gradient	*E*_*a*_ (kJ/mol)
10	163	0.997	− 0.382	7.333
20	178	0.997	− 0.376	7.236
30	185	0.998	− 0.364	6.947
40	169	0.991	− 0.531	10.131
50	143	0.992	− 0.739	14.183

**Figure 4:**
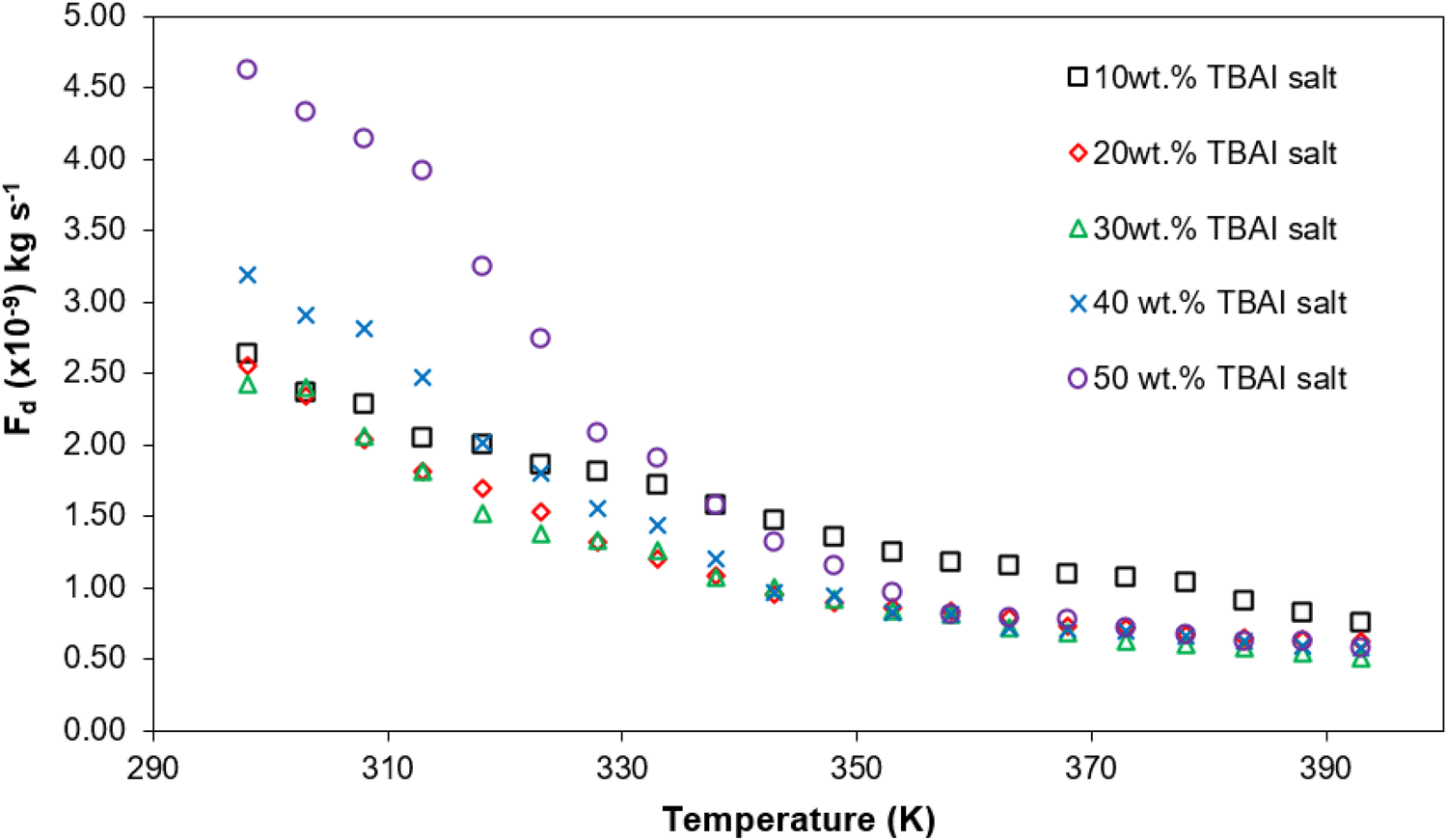
Stoke drag, $\mathop F\nolimits_{d}$ for various TBAI salt concentrations at selected temperature.

This is because at higher TBAI salt concentrations above 30 wt% TBAI salt, the mobility of charge carriers starts to decrease. As seen in Fig. 4, the number density of the charged carriers increased with the increasing concentration of TBAI salt in the PUA gel matrix. This means more free ions are present in the polymer matrix which in return increases the rate of collision between TBA^+^ cations and I^−^ anions.

The higher rate of collisions between the cations and anions will result in a higher chance of them forming a neutral charged pair. In other words, the TBAI salt prefers to remain in its salt form and not in its dissociated ion pair form. This will indirectly result in reduced mobility charged carriers and the polymer chain will become rigid^47,48^ as well as the kinetic energy of the ions will be reduced. This causes the free ions in the PUA polymer to move with difficulty and required more energy. Thus, high activation energy was required. This is supported by Fig. 3 and Table 3.

Apart from this, the stoke drag, $\mathop F\nolimits_{d}$ can be calculated from the value of the diffusion coefficient of the charge carriers that was obtained from fitting the Nyquist plots (Fig. 3). The Stoke drag value for various TBAI salt concentrations at selected temperatures is shown in Fig. 4. The stoke drag result showed an opposite trend compared to the diffusion coefficient of the charge carriers. The Stoke drag value for 30 wt% TBAI salt was the lowest as shown in Fig. 4 while the same salt concentration recorded the highest value of diffusion coefficient as shown in Fig. 3. This inverse relationship of the stoke drag and diffusion coefficient can also be seen in Eq. (10). Other than that, the sample containing 30 wt% TBAI salt required less energy to move as it had the lowest activation energy of 6.947 kJ/mol compared to other samples as shown in Table 3. The decrease of the calculated Stoke drag value could mean that the viscosity was lower. An electrolyte system with a lower viscosity will result in the free ions to move more readily. The Stoke drag value could also be related to the increase in the degree of amorphousness of the polymer matrix.

### Dielectric studies

To further understand the ionic conductivity behaviour in PUA-TBAI-I_2_ GPE, the complex permittivity was studied. The two main components in complex permittivity are the dielectric constant ($\varepsilon_{r}$) and dielectric loss ($\varepsilon_{i}$). The dielectric constant $\varepsilon_{r}$ is a measurement of the electric charge stored in the material^49,50^ while $\varepsilon_{i}$ is a measurement of energy loss during the electric polarity^51,52^.

**Figure 5:**
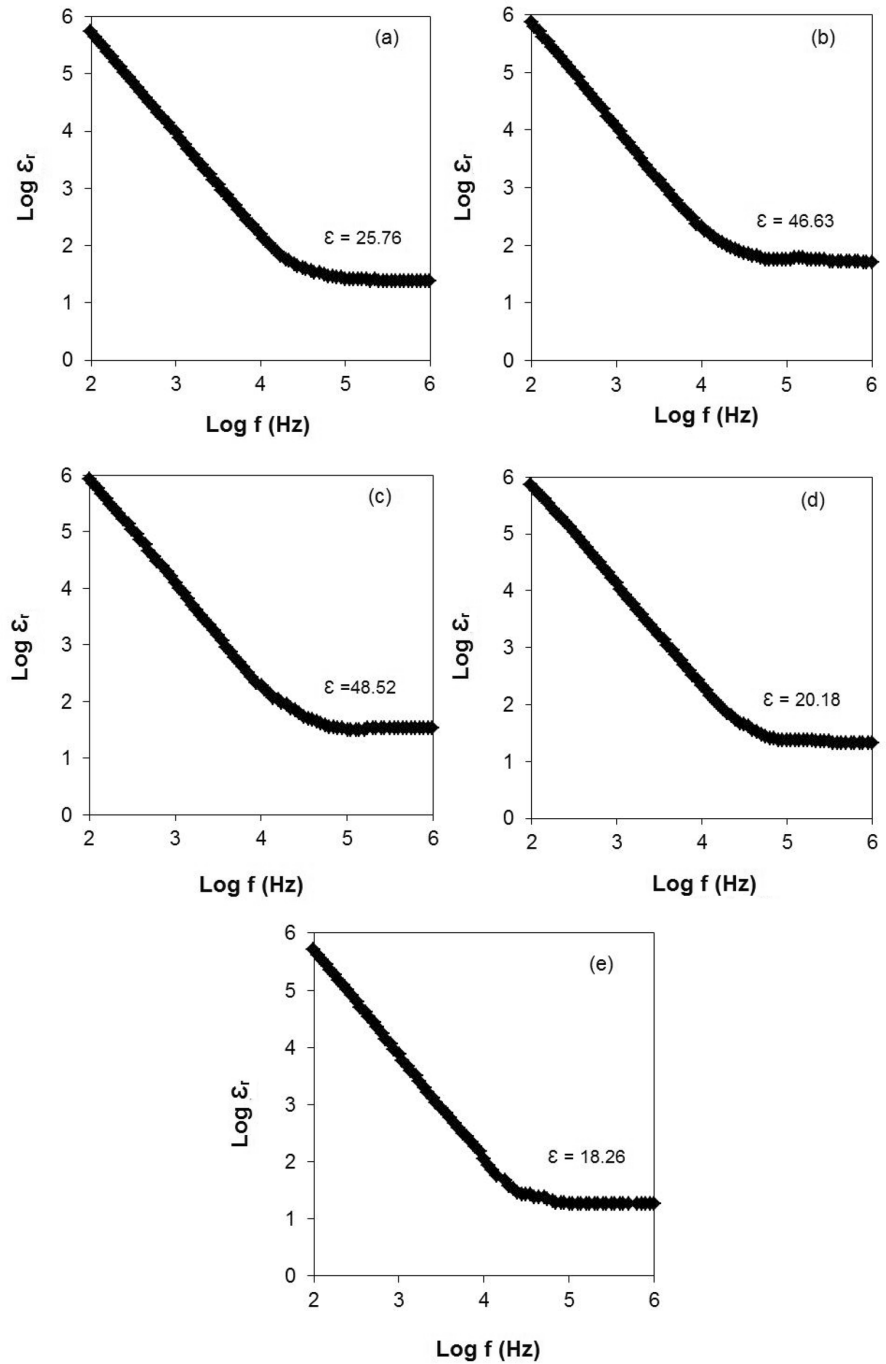
Log $\varepsilon_{r}$ (Log dielectric constant) against log frequency at room temperature for PUA GPE with (**a**) 10 wt%, (**b**) 20 wt%, (**c**) 30 wt%, (**d**) 40 wt% and (**e**) 50 wt% of TBAI salt.

Figure 5 shows a plot for log $\varepsilon_{r}$ against log frequency at room temperature (298 K) for PUA-based GPE with various TBAI salt concentrations. From Fig. 5, the $\varepsilon_{r}$ increased as the concentration of TBAI salt increased. This is because of an increase of free ions present in the PUA-based electrolyte^53^. As more free ions became present, more charges could accumulate at the border surface of the electrolyte/electrode as tracked by the non-Debye method. Thus, the amount of stored charge increased^54^ and indirectly, it enhanced the ionic conductivity as shown in Table 2. This can be proved by Ramesh et al*.*^55^. They stated that the increase of the dielectric constant of the plasticised system (PMMA-LiCF_3_SO_3_-dibutyl phthalate) was assigned to increase the charge carrier density. Another example was the addition of ethylene carbonate (EC) into PVA-LiBr-H_2_SO_4_ by Sheha^56^. The author said that the increase of dielectric constant was able to increase the storage charge in the electrolyte system. This was due to the ion dissociation in the EC plasticised polymer system as EC has a higher dielectric constant value and it causes more salts to be dissociated. It is best to described why $\varepsilon_{r}$ increases from 25.76 for 10 wt% TBAI to 48.52 for 30 wt% TBAI salt. For the excess 30 wt% TBAI salt, the $\varepsilon_{r}$ started to drop. This was due to the effect of ion polarisation^51,57^. Ion polarisation occurring with the formation of ion pairs is more favourable compared to the formation of free ions. When free ions are in an excess amount, the free ions will begin to interact with each other and form neutral charge pairs. Therefore, the mobility as well as ionic conductivity will reduce as shown in Table 2. This was able to explain the slump of $\varepsilon_{r}$ beyond the 30 wt% TBAI salt.

**Figure 6:**
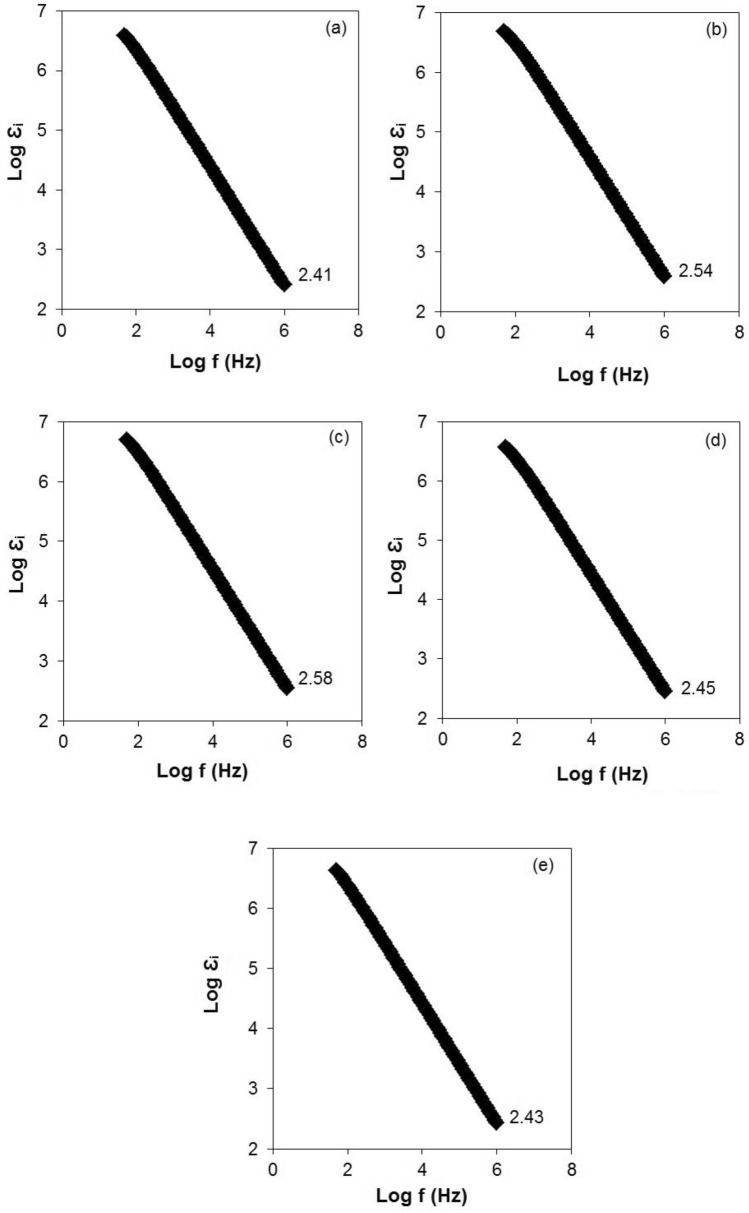
Log $\varepsilon_{i}$(Log dielectric loss) versus log frequency for (**a**) 10 wt%, (**b**) 20 wt%, (**c**) 30 wt%, (**d**) 40 wt% and (**e**) 50 wt% of TBAI salt.

Furthermore, a graph of log dielectric loss (log $\varepsilon_{i}$) versus log frequency at different concentrations of TBAI salt is shown in Fig. 6. At the lower frequency, the $\varepsilon_{i}$ is high due to the high number density of free ions. In this case, the charges accumulated at the electrolyte/electrode interface will build up as it has enough time to pile up at the interface before the electric field reverses. Besides that, $\varepsilon_{i}$ declined linearly as the frequency increased and this was attributed to the high periodic reversal of the electric field at the electrolyte/electrode interface^58^. This meant that no extra ion diffusion occurred in the direction of the electric field as there was not enough time to accumulate at the electrolyte/electrode interface^59^. Under those circumstances, the $\varepsilon_{i}$ dropped as the concentration of TBAI salt increased. In Fig. 6, the sample containing 30 wt% TBAI salt showed the highest $\varepsilon_{i}$ value. The $\varepsilon_{i}$ value for 10 wt% TBAI salt was 2.41 and it increased to 2.58 for 30 wt% TBAI salt. Excess beyond 30 wt% TBAI salt recorded a minimum $\varepsilon_{i}$ value which was 2.43 for 50 wt% TBAI salt.

**Figure 7:**
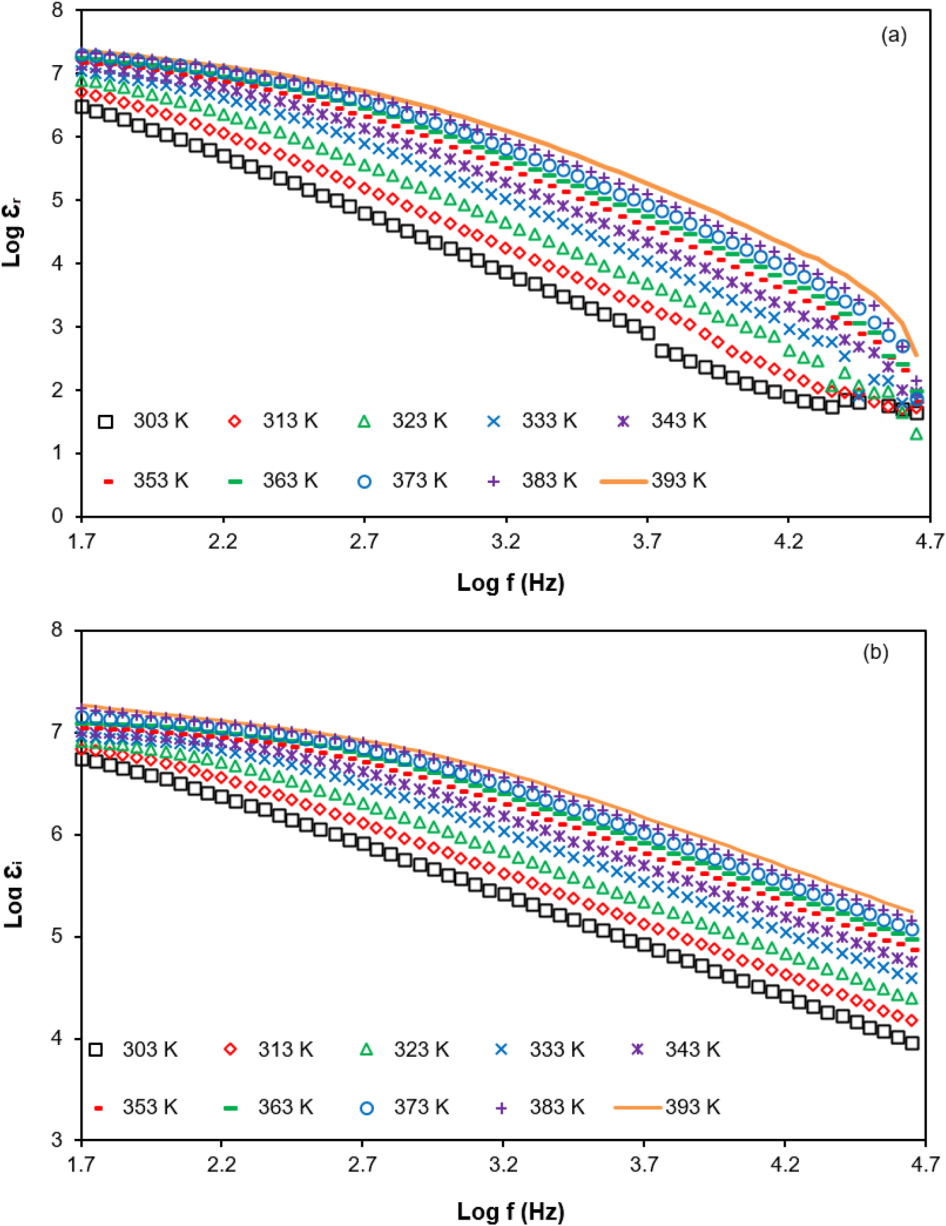
(**a**) Dielectric constant and (**b**) dielectric loss for PUA-30 wt% TBAI-I_2_ GPE at selected temperatures.

The dielectric properties can vary due to the frequency applied, temperature, structural characteristics or other factors^60,61^. In this part, the dielectric properties at various temperatures for PUA-30 wt% TBAI-I_2_ GPE (the highest ionic conductivity at room temperature) is discussed. Figure 7a,b display the dielectric properties of PUA-30 wt% TBAI-I_2_ GPE at selected temperatures (303 K to 393 K). It can be observed clearly that both $\varepsilon_{r}$ and $\varepsilon_{i}$ increased with increasing temperature. The increase of $\varepsilon_{r}$ and $\varepsilon_{i}$ may describe the increase of the number density of free ions as the temperature increased (recorded in Fig. 3). When the ions receive sufficient energy, they will begin to break and separate from each other to form free cations and anions. These lead to more free ions present in the electrolyte system. The circumstance can be related to Eq. (18) as below^53,62^:

18 $$n = n_{o} \exp \left( { - \frac{U}{{\varepsilon_{r} k_{b} T}}} \right)$$

Here *n* is charge density, *U* is dissociation energy, *k*_*b*_ is the Boltzmann constant and *T* is the absolute temperature. From Eq. (18), it is well known that the value of $\varepsilon_{r}$ depends on the charge concentration and temperature. The climb up of the number density of free ions with temperature will enhance the ionic conductivity as correlated to Eq. (16). Other than that, the rise of both $\varepsilon_{r}$ and $\varepsilon_{i}$ is due to the increased mobility. As the temperature increased, the ions will accelerate and move freely to attach with an electronegative group and indirectly they are able to enhance the ionic conductivity.

**Figure 8:**
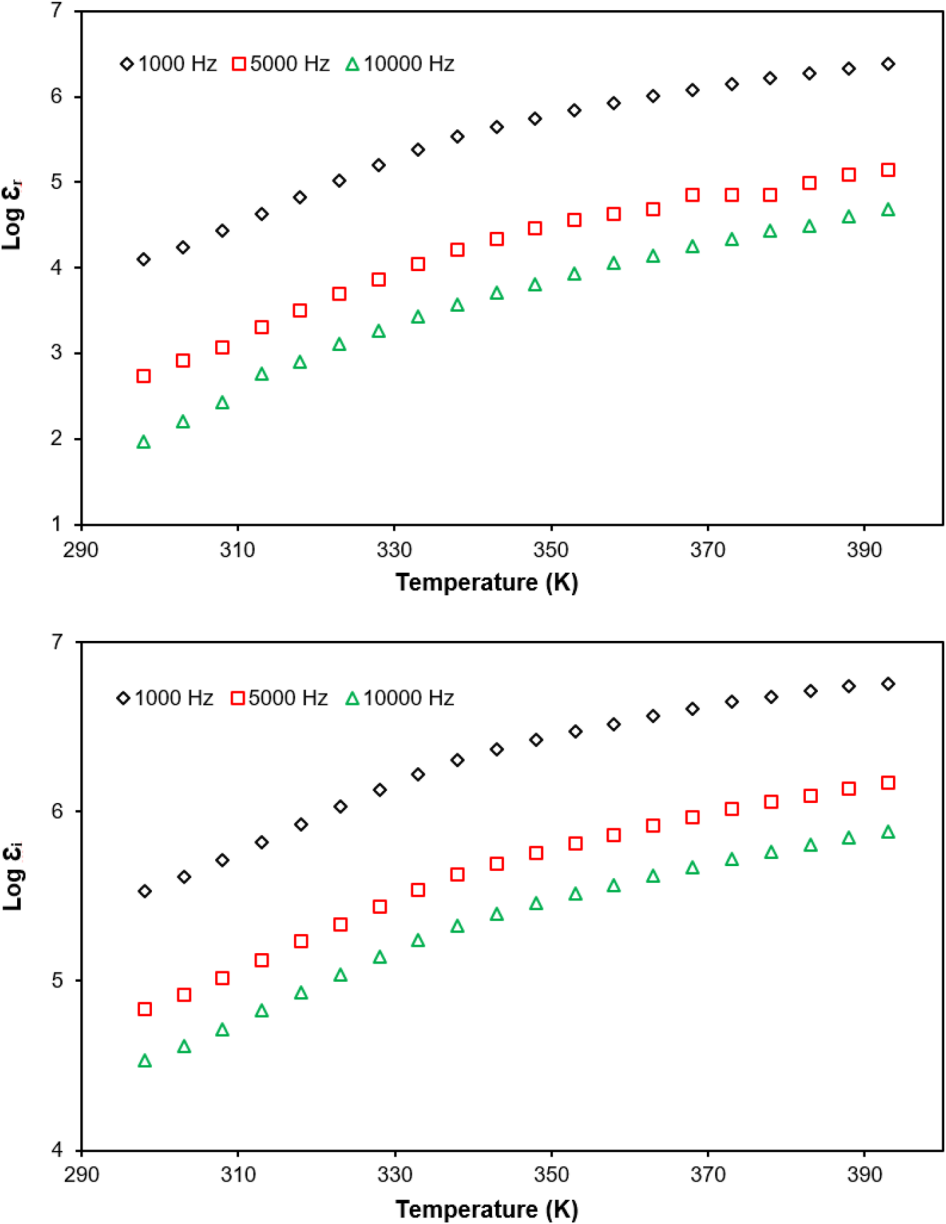
(**a**) Dielectric constant and (**b**) dielectric loss for PUA-30 wt% TBAI-I_2_ GPE at selected frequencies.

Figure 8 shows the graphs of the log dielectric constant and log dielectric loss against temperature at selected frequencies which were 1 kHz, 5 kHz and 10 kHz for PUA-30 wt% TBAI-I_2_ GPE. Based on the Fig. 8, it was observed that $\varepsilon_{r}$ and $\varepsilon_{i}$ decreased with increasing frequency. This can be explained by using the Maxwell–Wagner model. When the electric field was applied, it produced a space charge polarisation within the system. The space charge polarisation depends on the charge carriers and ionic conductivity of the sample. The lowest dielectric constant and dielectric loss value at high frequency was due to the fast reversal of the electric field^61^. At the highest frequency, the ion dipoles were unable to respond fast enough to the applied electric field in sufficient time. Thus, the ion-ion interaction tended to decrease. Hence, $\varepsilon_{r}$ and $\varepsilon_{i}$ increased at low frequency and decreased with increasing frequency.

### Modulus studies

**Figure 9:**
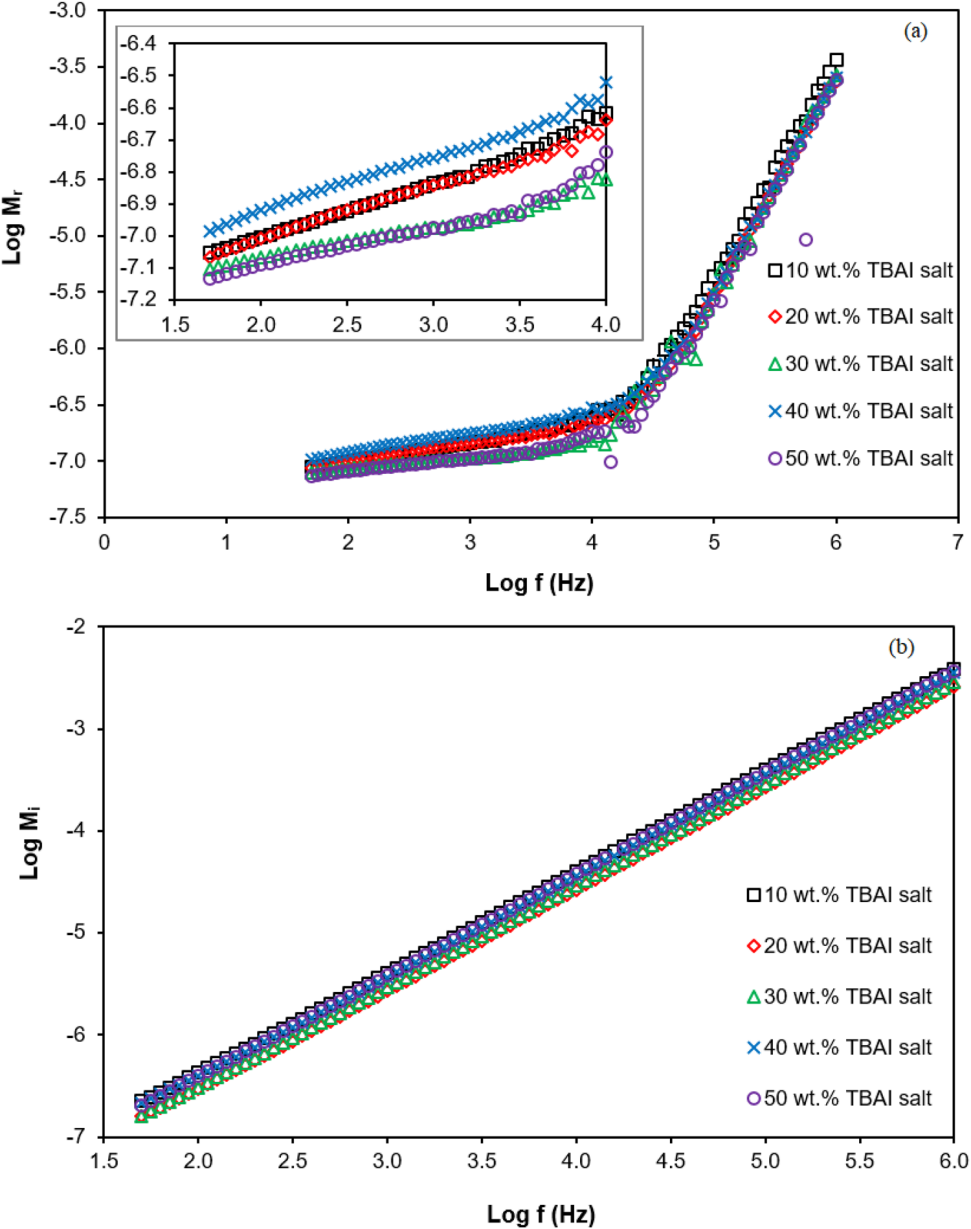
Variation of real ($M_{r}$) and imaginary ($M_{i}$) of electrical modulus with frequency for PUA-TBAI-I_2_ GPE system.

To deeply understand the dielectric behaviour of PUA-TBAI-I_2_ GPE, the complex electrical modulus was studied. Figure 9 shows the respective plots of $M_{r}$ and $M_{i}$ as a function of frequency for the PUA-TBAI-I_2_ GPE system. According to Fig. 9, $M_{r}$ and $M_{i}$ increased as the frequency increased. At lower frequencies, the values of $M_{r}$ and $M_{i}$ were kept constant at zero value in the frequency range from 50 Hz to 100 kHz. The presence of this long tail was due to the large capacitance associated with the electrodes^49,62–64^ and caused the removal of electrode polarisation as the effect was very small until it could be ignored^63^. This was because of the number density of free ions increased as the TBAI salt concentration increased (proved in Table 2). Additionally, beyond 32 kHz ($M_{i}$ plot) and 100 kHz ($M_{r}$ plot), both $M_{r}$ and $M_{i}$ increased gradually with increased frequency. This increase of $M_{r}$ and $M_{i}$ at high frequency indicated that the PUA-TBAI electrolytes were ionic conductors^65^.

**Figure 10:**
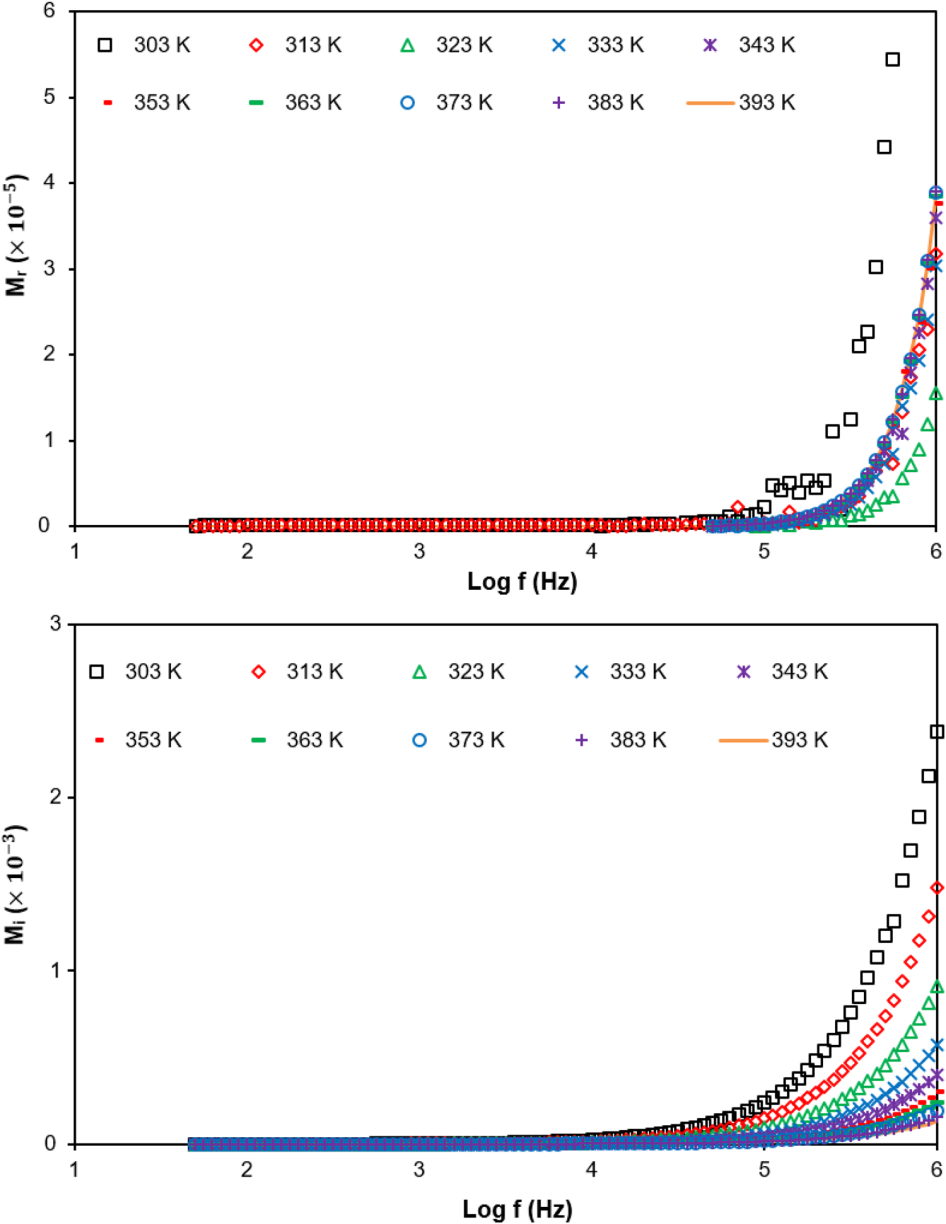
Real and imaginary modulus at various temperatures for PUA-30 wt% TBAI-I_2_ GPE.

Figure 10 shows the $M_{r}$ and $M_{i}$ for the highest ionic conductivity of PUA-30 wt% TBAI-I_2_ GPE electrolyte at various temperatures in the range of 303 K until 393 K. The long tail at lower frequency shows that there were no relaxation peaks. Figure 10 shows that the peak of $M_{i}$ shifted toward a higher frequency as the temperature increased. As the peak of $M_{i}$ shifted toward a higher frequency with increasing temperature, it indicated that the relaxation time decreased (supported in Fig. 13). This was because as the temperature increased, the aggregate ions tended to re-dissociate and cause more free ions to be produced. Further, the mobility of the free ions increased and hence, the ionic conductivity could be improved.

### Capacitance

Based on the Fig. 9 for $M_{i}$ versus log frequency, the capacitance of PUA-TBAI-I_2_ GPE may be calculated using Eq. (19).

19 $$C = \frac{{\varepsilon_{o} }}{{2M_{{i\left( {\max } \right)}} }}$$

Table 4 shows the capacitance values for various concentrations of TBAI salt. The 30 wt% TBAI salt produced the largest capacitance value of 14.6 pF. The capacitance obtained was in the range of 2 to 20 pF and was due to bulk conduction. This might be due to the increase of the number density in the PUA-TBAI-I_2_ GPE system as the concentration of the TBAI salt increased. When the number density of the charge carriers increased, more free ions became present in the electrolyte system and they moved faster to reach their destination. Furthermore, it could be observed that a large capacitance had a greater value in terms of dielectric constant as seen in Fig. 5. This was because the dielectric constant was a property of the dielectric material. For this reason, PUA-30 wt% TBAI-I_2_ GPE recorded the highest energy stored as it had a greater value in terms of dielectric constant and capacitance. The same was true in the research done by Ramesh et al.^55^. Furthermore, as more free ions became present in the electrolyte system, the ion-ion interaction formed and caused the mobility of the ions to reduce and hence more neutral charge became present. This could lead to a decrease in the value of the dielectric constant and indirectly affected the energy stored in the PUA-TBAI-I_2_ GPE system. Due to that, the capacitance value beyond 30 wt% TBAI salt decreased.

**Table 4:** Measured capacitance for various wt% of TBAI salt.

TBAI (wt%)	$M_{{i\left( {\max } \right)}}$(× 10^–3^)	Capacitance (× 10^–11^ F)
10	3.84	1.15
20	3.08	1.44
30	3.03	1.46
40	3.53	1.25
50	3.69	1.20

For temperature dependence, the capacitance can be calculated by:

19 $$C\, = \,\frac{1}{{k_{2}^{ - 1} \,}}$$

**Figure 11:**
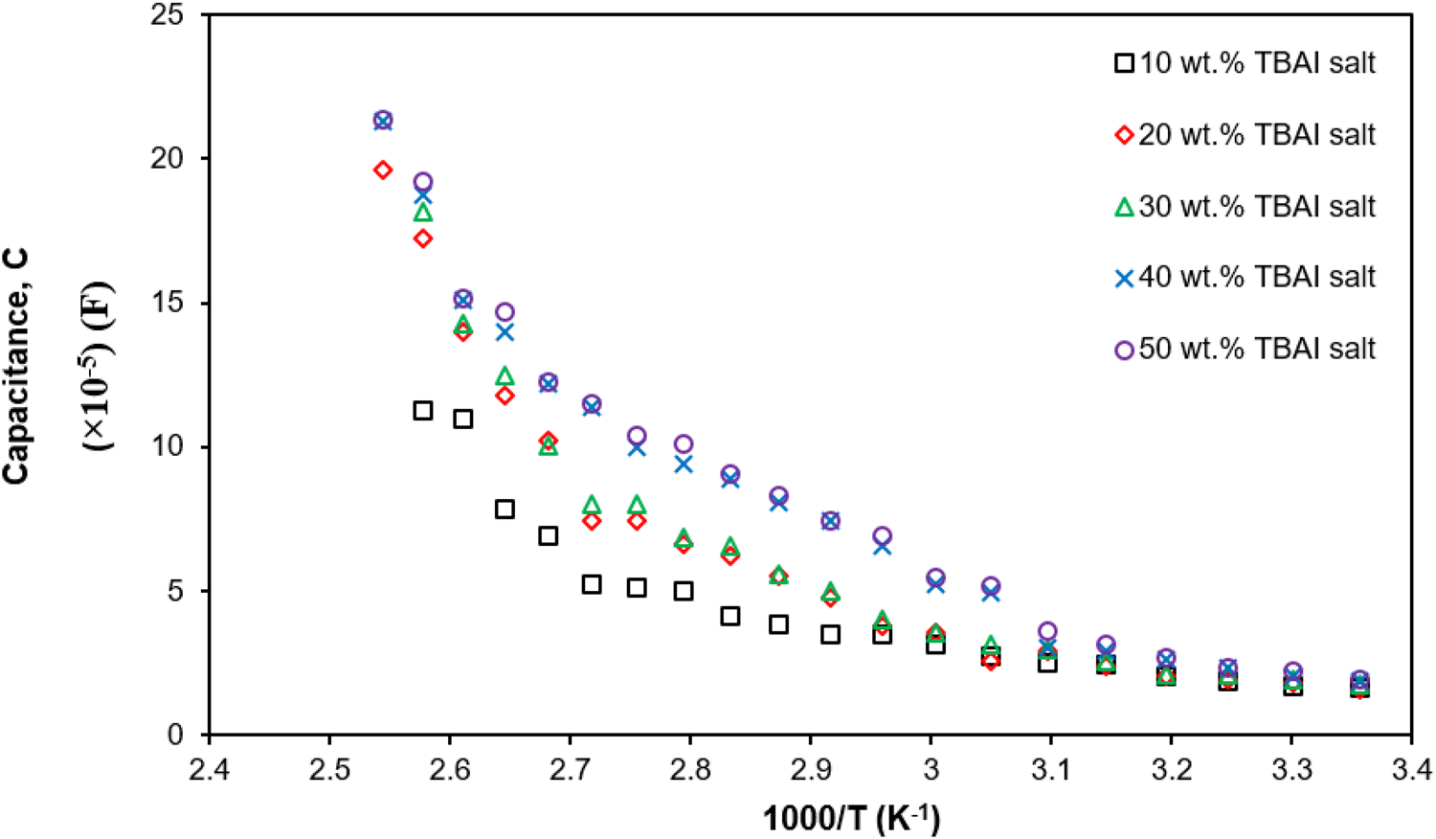
Temperature dependence of capacitance at different temperatures.

Here ${k}_{2}^{-1}$ is the capacitance due to the Electric Double Layer (EDL) formed at the electrode/electrolyte interface during the impedance measurement. The value of ${k}_{2}^{-1}$ was obtained by trial and error until the Nyquist plot fitted correctly. Based on Fig. 11, it can be clearly seen that the capacitance increased with the increasing concentration of TBAI salt as well as the temperature increase. This is to say when the current flowed into the capacitor, it started to build a charge and the charge number would increase as shown in Fig. 3^66^. Then, the charge gathered at the electrode/electrolyte interface and in return caused the electrostatic field to become stronger. Thus, more energy was able to be stored in the electrolyte. This suggested that the capacitance value would increase.

### Loss tangent

**Figure 12:**
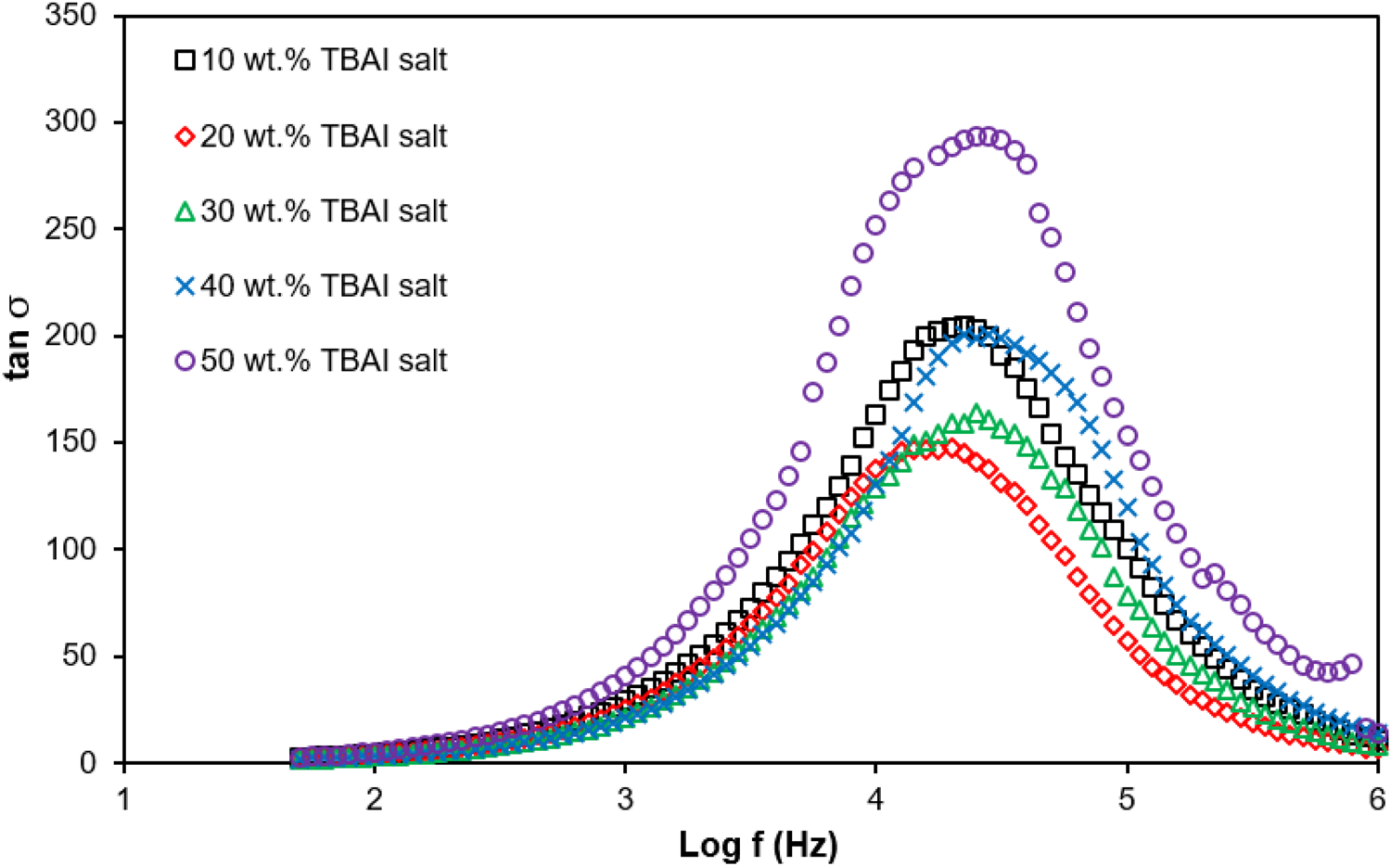
tan σ versus log frequency for PUA-TBAI-I_2_ GPE system at room temperature (298 K).

Figure 12 shows the graph of tan *δ* versus log frequency for the PUA-TBAI-I_2_ GPE system at room temperature (298 K). The relaxation time of PUA-TBAI-I_2_ GPE systems are tabulated in Table 5 by referring to the maximum peak of tan *δ*. As the peak shifted toward higher frequencies, it was observed that the relaxation time was reduced^67^. From Fig. 12, it can been seen that the 30 wt% TBAI salt peak shifted towards a higher frequency compared to other samples. In other words, it implies that the free ions in the 30 wt% TBAI salt system were moving rapidly and the relaxation time was shorter and because of that the ionic conductivity was enhanced. Above 30 wt% TBAI salt, the relaxation time taken was observed to be slower which also registered a reduction in ionic conductivity. Hence, 30 wt% TBAI salt showed that it had the shortest relaxation time (3.98 × 10^–5^ s) as well as highest in ionic conductivity which was (1.88 ± 0.020) × 10^–4^ S cm^−1^ at room temperature.

**Table 5:** Relaxation time for PUA-TBAI-I_2_ GPE system at room temperature.

TBAI (wt%)	τ (× 10^–5^ s)
10	5.62
20	5.01
30	3.98
40	4.47
50	5.01

**Figure 13:**
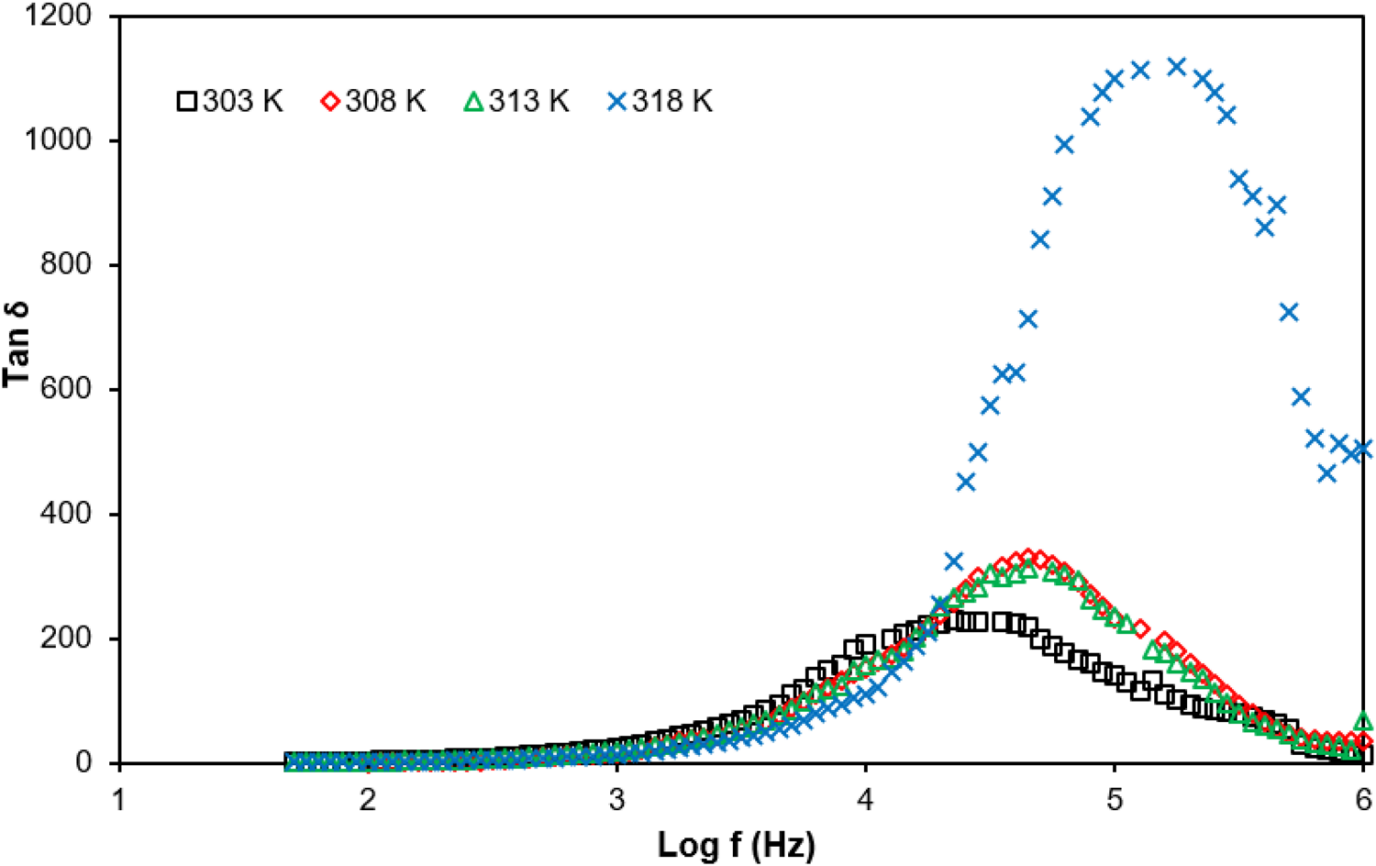
Frequency dependence of tan δ for PUA-30 wt% TBAI-I_2_ GPE at selected temperatures. Shifting the peak towards higher frequency indicated the increase of ionic conductivity.

Figure 13 shows a graph of tan σ for PUA-30 wt% TBAI -I_2_ GPE at selected temperatures and relaxation time at Table 6. According to Fig. 13, it indicates that tan δ increased with frequency. After reaching the maximum then it started to decrease. Furthermore, the peaks were shifted to a higher frequency as the temperature increased from 303 to 318 K. This explains the shorter relaxation time. In Fig. 13, it shows the relaxation time was reduced from 4.47 × 10^–5^ s at a temperature of 303 K to 0.56 × 10^–5^ s at a temperature of 318 K. Hence, this proved that the relaxation time was reduced as the temperature increased.

**Table 6:** Relaxation time for PUA-30 wt% TBAI-I_2_ GPE system at selected temperatures.

Temperature (K)	τ (× 10^–5^ s)
303	4.47
308	2.24
313	2.00
318	0.56

## Conclusion

In this study, jatropha oil based PUA gel polymer electrolyte enhanced with TBAI-I_2_ showed promising results and that it was feasible to be used as an alternative green and sustainable electrolyte. The GPE system also managed to perform on par with other green and sustainable polymer electrolytes. The 30 wt% TBAI recorded the highest ionic conductivity at all temperatures tested, starting from room temperature of 298 K up to 393 K compared with the other ratios prepared in this work. This showed that a high amount of TBAI salts were not necessary to obtain greater ionic conductivity as there were diminishing returns beyond the 30 wt% TBAI. The mobility and diffusion coefficient of the charge carriers also corresponded to the positive result of the ionic conductivity. The 30 wt% TBAI also reported the lowest pseudo-activation energy compared to other samples at 6.947 kJ mol^−1^. The values of the dielectric constant, dielectric loss, electrical modulus, dissipation factor, stoke drag and the capacitance were also reported in this work accordingly and affirmed the great performance of the 30 wt% TBAI-PUA polymer in terms of electrochemical properties.
